# A Data Fusion Modeling Framework for Retrieval of Land Surface Temperature from Landsat-8 and MODIS Data

**DOI:** 10.3390/s20154337

**Published:** 2020-08-04

**Authors:** Guohui Zhao, Yaonan Zhang, Junlei Tan, Cong Li, Yanrun Ren

**Affiliations:** 1 Science Big Data Center of Cold and Arid Regions, Northwest Institute of Eco-Environment and Resources, Chinese Academy of Sciences, Lanzhou 730000, China; renyr@lzb.ac.cn; 2 University of Chinese Academy of Sciences, Beijing 100049, China; shuimulicong@lut.edu.cn; 3 Heihe Remote Sensing Experimental Research Station, Northwest Institute of Eco-Environment and Resources, Chinese Academy of Sciences, Lanzhou 730000, China; tanjunlei@lzb.ac.cn; 4 College of Computer and Communication, Lanzhou University of Technology, Lanzhou 730050, China

**Keywords:** land surface temperature, joint retrieval, data fusion, MODIS, Landsat-8

## Abstract

Land surface temperature (LST) is a critical state variable of land surface energy equilibrium and a key indicator of environmental change such as climate change, urban heat island, and freezing-thawing hazard. The high spatial and temporal resolution datasets are urgently needed for a variety of environmental change studies, especially in remote areas with few LST observation stations. MODIS and Landsat satellites have complementary characteristics in terms of spatial and temporal resolution for LST retrieval. To make full use of their respective advantages, this paper developed a pixel-based multi-spatial resolution adaptive fusion modeling framework (called pMSRAFM). As an instance of this framework, the data fusion model for joint retrieval of LST from Landsat-8 and MODIS data was implemented to generate the synthetic LST with Landsat-like spatial resolution and MODIS temporal information. The performance of pMSRAFM was tested and validated in the Heihe River Basin located in China. The results of six experiments showed that the fused LST was high similarity to the direct Landsat-derived LST with structural similarity index (*SSIM*) of 0.83 and the index of agreement (*d*) of 0.84. The range of *SSIM* was 0.65–0.88, the root mean square error *(RMSE)* yielded a range of 1.6–3.4 °C, and the averaged *bias* was 0.6 °C. Furthermore, the temporal information of MODIS LST was retained and optimized in the synthetic LST. The *RMSE* ranged from 0.7 °C to 1.5 °C with an average value of 1.1 °C. When compared with in situ LST observations, the mean absolute error and *bias* were reduced after fusion with the mean absolute bias of 1.3 °C. The validation results that fused LST possesses the spatial pattern of Landsat-derived LSTs and inherits most of the temporal properties of MODIS LSTs at the same time, so it can provide more accurate and credible information. Consequently, pMSRAFM can be served as a promising and practical fusion framework to prepare a high-quality LST spatiotemporal dataset for various applications in environment studies.

## 1. Introduction

Land surface temperature (LST) is the measurement of the radiative skin temperature over the earth’s surface [1]. As a crucial variable associated with the energy balance, LST is highly responsive to land surface energy equilibrium [2,3], which may contribute to explore a variety of phenomena taking place at the surface-atmosphere interface [4]. Therefore, it becomes valuable for various scientific studies [1], such as climatology [5], drought monitoring [6], urban heat island [7,8,9], hydrology [10,11], infrastructure [12], agriculture [13,14], public health [15], and permafrost mapping [16]. However, the remote areas have limitations of using in situ automatic meteorological stations because it is cost-intensive due to involved instrumentation and maintenance, which makes the spatial continuity of data-sparse [17]. Especially, in remote mountainous areas where the in situ measurements are of scarcity and uneven, satellite-derived LSTs can serve as an efficient proxy for air temperature estimation [18]. Thus, understanding and monitoring the dynamics of LST and its links to human-induced changes are critical for modeling and predicting environmental change and climate variability [19].

LST varies significantly over time and space, while the ground LST measurements can only provide the observation for the surrounding area of influence within the observation stations of view [4,20]. The LSTs derived from Landsat, MODIS, AVHRR, and other thermal infrared (TIR) satellites have been widely and effectively used to monitor and understand LST changes at regional and global scales [21,22], particularly in remote and large areas. The satellite-derived LSTs are suited equally to both quick pilot studies as well as long-term monitoring [5], which are publicly available for characterizing the spatiotemporal variations of the thermal condition at spatial resolutions of 60 m to 10 km and temporal resolutions of hour to month [23]. The most common LST products are those derived from the MODIS instruments on board of Terra and Aqua satellites, which have been popularly used all over the world [20]. However, due to technical constraints, the existing satellite platforms have a trade-off between temporal and spatial resolutions. The higher spatial resolution of the satellite sensor causes swath observation to be narrower and thus exceeds the revisit time, and vice versa [24]. As a result, none of the available satellites can provide concurrently high spatial resolution and high temporal resolution products [25], which significantly restricts the potential application of satellite-derived LST in various fields [26,27,28,29]. As a result, a high-quality LST dataset is urgently needed for monitoring the thermal dynamics with high spatial and temporal variability [2,20,30].

Data fusion is an effective way to improve the temporal and spatial resolution of satellite-derived data products. Thus, many scholars have been committed to developing various techniques to enhance the spatial and temporal resolution of remote sensing data [2,31,32,33]. Existing techniques have been named differently [34,35]. In this study, we used ‘fusion’ as defined by Zhang et al. [34]. To be specific, multi-sensor data fusion is a synthesis processing of making full use of the spatial details of high spatial resolution (HSR) data to integrate the temporal information of low spatial resolution (LSR) data to generate a synthetic data product of which information is more credible and more reliable than that of a single sensor source [34]. Intrinsically, it is also a form of downscaling for LSR data, because the fused data has a spatial pattern of HSR data and preserves the temporal attribute of LSR data. According to the results of fusion, these existing techniques can be broadly grouped into spatial fusion and spatiotemporal fusion. The spatial fusion techniques have been largely developed to disaggregate LSR LST to HSR LST based on HSR auxiliary dataset. For example, the Pixel Block Intensity Modulation (PBIM) method was proposed by Liu and Moore [36] to improve the spatial resolution of the Landsat-5 thermal band from 120 m to 30 m by adding spatial detail of topographic variations. Then, the modulation method has been used to fuse the brightness temperature of ASTER with SPOT-5 [37] and downscale AVHRR LSTs into Landsat-5 pixel size by employing different scaling factors [38]. Besides, there are some methods for single-sensor TIR data including Generalized Laplacian Pyramid [39], Bayesian fusion approach [39], Co-kriging interpolation [40], and TsHARP [41]. The spatial fusion techniques are designed to allocate LSR pixel value to each HSR pixels, and the allocation factors usually include ratio, topography, normalized difference vegetation index (NDVI), and emissivity. However, some methods ignore the physical basis of TIR remote sensing and spatial dependence and heterogeneity [42]. The spatiotemporal fusion simultaneously enhances the temporal and spatial resolution of TIR data for a comprehensive analysis of the thermal variations. Gao et al. [43] proposed the Spatial and Temporal Adaptive Reflectance Fusion Model (STARFM) to generate daily synthetic surface reflectance images at 30 m spatial resolution. As a widely used data fusion model, it was later adjusted and revised for specific applications under different scenarios [11,15,27,44,45,46]. Although widely used, some critical issues of STARFM and its variants applied to LST prediction have not been solved [30,47], such as the need for more HSR and LSR imagery pairs, the complexity of calculation, and the dynamics of the land surface was treated statically or linearly [25,28,35]. As for LST fusion, they are mainly from the perspective of surface reflectance [27,29,45,48,49], and few studies formulate a common framework for generating synthetic LST products.

To date, a variety of fusion methods have been proposed to achieve both high spatial and temporal resolutions based on the combination of Landsat and MODIS [50,51]. MODIS and Landsat-8 have complementary characteristics in terms of spatial and temporal resolution, especially the two TIR bands. The two TIR channels of Landsat-8 are band 10 and band 11, and the central wavelengths are 10.9 μm and 12 μm, respectively, which are very similar to Band 31 and Band 32 of MODIS. It lay the foundation for the LST fusion of these two satellites at the product level. MODIS LST products are excellent in temporal resolution, and the spatial resolution is adequate to study the global LST variations, but it may not be appropriate for regional research [52]. On the contrary, the 30 m spatial resolution, multispectral bands, and freely available data constitute an excellent utility for a wide range of applications based on Landsat satellites, which provide sufficient spatial details for monitoring land surface changes at the regional scale [53]. However, the 16-day revisit-cycle, even longer because of inferior atmospheric conditions, has limited further applications [51,54]. Beyond that, it is relatively difficult to automatically retrieve accurate and valid LST from Landsat TIR bands, although there are several algorithms available [21,55]. When using these direct retrieval algorithms, the preparation of the corresponding parameters and auxiliary data (such as emissivity, atmospheric parameters, etc.) is rather complicated and tedious [50]. Meanwhile, there is not yet a universal LST product derived from Landsat-8. It is a practical solution to combine MODIS and Landsat to learn more about the temporal and spatial variations of LST [9,30]. The common idea of these methods is to disaggregate LSR data into HSR data based on a pair of co-registered imageries to produce high spatiotemporal products. Each method has its own strengths and limitations, and some reviews on best practices of these methods were briefly described by Hazaymeh and Hassan et al. [47], Zhu et al. [2], and Zhao et al. [28].

The continuous monitoring of the LST dynamics is helpful in assessing the impact of climate change and human activities on environmental change. Thus, it is necessary to develop a practical method to generate the high-quality LST spatiotemporal dataset for exploring the spatial pattern and temporal variations by aggregating multi-source satellite data. Despite the fusion method progresses, the development of new techniques in the LST fusion should be further enhanced. In this paper, we mainly focus on how to generate a synthetic LST by blending the Landsat-8 TIR data and MODIS LST product. Specifically, a pixel-based multi-spatial resolution adaptive fusion modeling framework (named pMSRAFM) is proposed for generating the high-quality synthetic LST in an automated manner. The pMSRAFM uses MODIS LST as prior knowledge to solve the parameters of the Landsat-8 retrieval algorithm for generating LST at Landsat-like resolution on the MODIS observation time. In a sense, it is a disaggregation approach of MODIS LST products with the main difference in the decomposition of the mixed pixels. The remainder of this paper is organized as follows. Section 2 first describes the test region and the dataset used for experiments. Then, it presents a brief introduction to the fundamental theory and the details of the proposed framework. The results of comparison and validation are presented in Section 3. Finally, conclusions and suggestions for future work are provided in Section 4.

## 2. Materials and Methods

### 2.1. Study Area

**Figure 1:**
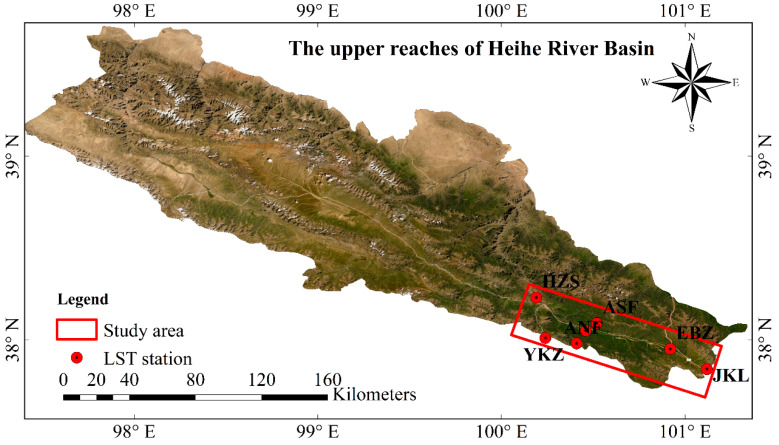
The study area (red rectangle line) and locations of land surface temperature (LST) stations.

The southeastern area (40°10′–42°00′ N and 100°10′–112°10′ E) of Heihe River Basin (HRB) was selected as the study area to test the performance of pMSRAFM (Figure 1). HRB is the second-largest endorheic basin with unique landscapes and coexisting cold and arid regions in northwestern China [56]. It features glaciers, frozen soil, alpine meadows, forests, irrigated crops, riparian ecosystems, and deserts from upstream to downstream [57]. The regional environment variation and monitoring investigation require the support of high spatial and temporal resolution LST datasets. The study area covers 3285 km^2^ and ranges in elevation from 2538 to 4615 m. As a climatic and geographical transition zone, the study area provides an ideal testing ground with complex land surface characteristics for verifying the rationality of the proposed LST fusion framework. Many scholars have verified the applicability of satellite-derived LSTs in this region, and the results showed that root mean square error (RMSE) was generally within 5K [42,58,59,60]. Besides, a large number of aeronautical experiments have been carried out in the HRB [61], and some ground observations can be provided to validate the fusion result and analyze the uncertainty. In situ observations were measured by the automatic TIR stations with an SI-111 infrared radiometer (8–14 µm). The information of seven LST stations operated by HiWATER [61] is described in Table 1 and displayed in Figure 1.

### 2.2. Data Collection and Processing

#### 2.2.1. Auxiliary Data

The auxiliary data can provide key information about spatial details to optimize the disaggregation and distribution of LSR pixels. The proper classification of HSR pixels is the most critical for the disaggregation of LSR pixels. For efficiency and convenience, pMSRAFM in this study assigns a unique type code to each HSR pixel based on the land cover category. Therefore, the accuracy of land cover data is the primary consideration. The FROM-GLC (Finer Resolution Observation and Monitoring-Global Land Cover, http://data.ess.tsinghua.edu.cn/) data was selected because of its excellent performance. FROM-GLC is the first 30 m resolution global land cover maps produced using Landsat Thematic Mapper (TM) and Enhanced Thematic Mapper Plus (ETM+) data [62]. The FROM-GLC data contains ten base classes (1-Cropland, 2-Forest, 3-Grassland, 4-Shrubland, 5-Wetland, 6-Water, 8-Impervious, 9-Bareland, 10-Snow and ice), and the overall accuracy of global land cover was above 65% [62]. Image refinement was executed to manually correct certain confusing pixels to further improve the classification accuracy. Since the latest FROM-GLC data only includes products for 2015 and 2017, the selection of satellite data was limited to these two years. The Shuttle Radar Topography Mission (SRTM) 1 Arc-Second Global elevation data was downloaded from the USGS EarthExplorer website (https://earthexplorer.usgs.gov/). These data were reprojected and resampled to match Landsat data.

#### 2.2.2. Satellite Data

The satellite data with minimal cloud cover and clear-sky conditions will be used to evaluate the proposed framework. Finally, Landsat-8 OLI/TIRS C1 Level-1 (L1TP) products of Path 133, Row 34, acquired on 13 September, 2015 (acquisition time 10:45 a.m.) and 16 July, 2017 (acquisition time 10:28 a.m.) were selected as the HSR data. As the highest quality Landsat data, the L1TP products are suitable for pixel-level time series analysis, which have been radiometrically calibrated and orthorectified using ground control points and DEM data to correct for relief displacement [63]. Besides, NDVI can be calculated based on the red and near-infrared bands of Landsat-8. Two types of MODIS daily LST products, MOD11_L2 version 6 swath product and the MOD11A1 version 6 product, were selected and used as the LSR LST data. These MODIS LST products provide daily per-pixel LST and emissivity with a 1 km spatial resolution. The MOD11_L2 is produced daily in 5-min temporal increments of satellite acquisition using the generalized split-window algorithm, and the MOD11A1 is derived from the MOD11_L2 swath product by projecting MOD11_L2 pixels to Earth locations on a sinusoidal mapping grid. These MODIS LST products and Landsat-8 L1TP data were collected from the USGS EarthExplorer. Note that the Landsat-8 TIRS bands provided by the USGS were resampled to 30 m to match multispectral bands. The MODIS products were converted and projected by the HDF-EOS to GeoTIFF Conversion Tool (HEG) [64] to the UTM projection the same as Landsat data.

To ensure the validity of the pixel values, quality control was performed on each input data. A shared quality assurance mask was created for both Landsat and MODIS data to select the valid pixels for the computation. First, a filter screened out the pixels that had the LST values beyond reasonable expectations according to the LST changes during that month. Second, quality flags from both MODIS and Landsat data were used to ensure that only high-quality pixels were selected and involved in fusion. The data quality of MODIS was controlled by built-in quality control flags. In practice, the selection criteria of MOD11A1 pixels was that the daytime LST quality indicators equal to 0. As for MOD11_L2, the LST error was constrained to less than 0.5 ℃. The data quality of Landsat-8 was controlled based on the Landsat Collection 1 Level-1 Quality Assessment 16-bit Band to select the clear pixels.

#### 2.2.3. Reference LST

To prepare HSR reference LST, we directly retrieved LST from Landsat-8 using the split-window algorithm (SWA) described by Du et al. [65] to obtain comparable high-quality reference LST ($LST_{HSR}^{SWA}$). The SWA could obtain LST with an accuracy of better than 1.0 k [65]. Furthermore, we prepared the second HSR reference LST ($LST_{HSR}^{SC}$) using the single-channel (SC) method based on Landsat-8 band 10 and FROM-GLC data. The Landsat-derived LSTs in the HRB have been verified with the in situ LSTs estimated from the flux measurements at HiWATER stations and the *RMSE* ranges from 1.12 °C to 3.67 °C [42,60]. These two HSR reference LSTs are complementary to each other, and both are useful to quantify and characterize the accuracy of the HSR fused LST. In addition, the corresponding observational records were collected from the LST stations located in the study area (Figure 1 and Table 1). These observational records were calibrated using the Stefan Boltzmann law as described in Duan et al. [22] to assess the accuracy of satellite-derived LSTs.

### 2.3. Methodology

**Figure 2:**
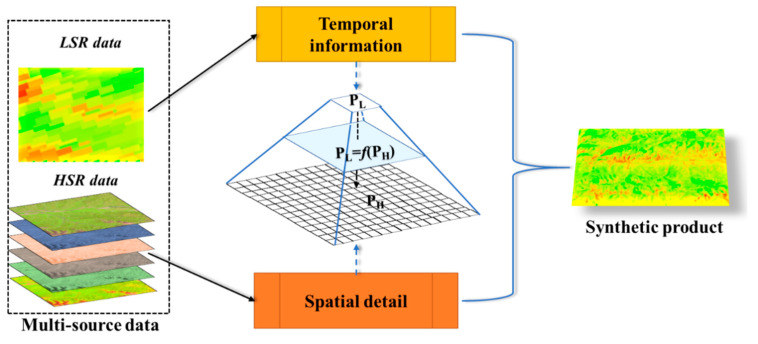
Diagram of the pixel-based multi-spatial resolution adaptive fusion modeling framework for multi-source data fusion.

In this study, two aspects are considered in advance when constructing the fusion framework. First, the fusion framework is expected to be mathematically simple, robust, and computationally efficient. Due to the fusion process requires ancillary data and is implemented for each pixel in sequential mode, it should be simple and efficient. The development of temporal-spatial fusion techniques has provided a foundation for establishing the basic framework for multi-source data fusion. The overview of the pMSRAFM framework for multi-source data fusion is described in Figure 2. Our proposed framework relies on the assumption that the LST derived from the different satellites with similar spectral bandwidth and wavelength in the same region at the same time should be highly consistent or linearly correlated. As a form of physical downscaling, the LSR pixel can be considered as an aggregated block of HSR pixels. That is, the LSR pixel value can be approximately expressed by a transfer function of a block of HSR pixel values. The pMSRAFM aims to minimize the global error and estimate an optimal transfer function by joint utilizing the spatiotemporal information derived from multi-source satellites. The solving process consists of three key steps. First, calculate the same physical variables from the multi-resolution data according to their respective retrieval algorithms and extract the multi-dimensional attributes from HSR input data as pixel features. Second, establish the corresponding relationship at the pixel-level between two kinds of data based on the characteristic of the land surface and automatically solve the equation that describes the corresponding relationship. Finally, apply the solved parameters to the retrieval algorithm of the HSR data to generate a new synthetic data product. The following uses LST fusion as a practical case to introduce the application of the pMSRAFM.

#### 2.3.1. LST Fusion with pMSRAFM

**Figure 3:**
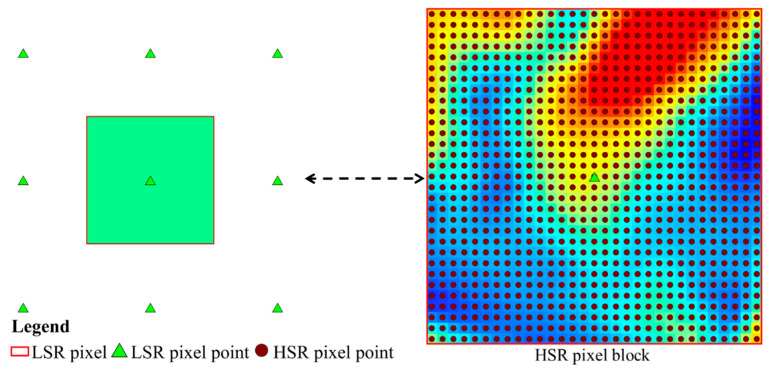
A schematic diagram of a low spatial resolution (LSR) pixel (green triangle) versus a block of high spatial resolution (HSR) pixels (dots with dark umber).

The basic idea of LST fusion based on the pMSRAFM is that LSR LST can be estimated by a weighted sum of the HSR LSTs. Essentially, the proposed LST fusion method is designed to integrate the temporal information from the LSR sensor with the spatial pattern of the HSR sensor to generate the high spatiotemporal LST data. It has been proved that different satellite-derived LSTs with different spatial resolutions exist a correlation at the low spatial resolution. In general, the LSR pixel can be seen as an aggregated block of the corresponding HSR pixels at the same time at the same location [35,66,67], as shown in Figure 3. So, the relationship between LSTs from two satellites over the homogeneous land surface can be expressed by the linear transfer function:

(1) $$LST_{LSR}\left(x,y,t\right)=f\left(LST_{HSR}\left(x,y,t\right)\right)=a\times LST_{HSR}\left(x,y,t\right)+b+\varepsilon \left(t\right)\;$$

where *LST_LSR_* and *LST_HSR_* define low-resolution LST and high-resolution LST, respectively. (*x*, *y*) represents a given location, and *t* is the acquisition time. *a* and *b* are the coefficients used to adjust the observational differences, ideally set to 1 and 0, respectively [44,51]. ε(t) is the residual representing the observational difference between these two satellite platforms. It is mainly due to the discrepancy in acquisition time, bandwidth, orbit parameters, geolocation errors, effective pixel coverage, and spectral response function. To handle the heterogeneous characteristics, we employ a similar method to the linear spectral mixture analysis [66,67,68] to distribute the temporal information of *LST_LSR_* according to the spatial details of *LST_HSR_*. Thus, each HSR pixel may have different parameters of *a* and *b*. Then the Equation (1) is reformed as:

(2) $$LST_{LSR}\left(t\right)=a_{1,t}\left[\frac{1}{N}\overset{N}{\underset{i=1}{\sum }}\left(a_{2,i}LST_{HSR,i}\left(t\right)+b_{2,i}\right)\right]+b_{1,t}+\varepsilon \left(t\right)\;$$

where $LST_{LSR}\left(t\right)$ is LSR pixel value at time t. $a_{1,t}$ and $b_{1,t}$ are regression coefficients between two satellites of the first layer at coarse resolution. *N* is the total number of HSR pixels corresponding to an LSR pixel. $LST_{HSR,i}\left(t\right)$ is the i-th HSR pixel value at time *t*. $a_{2,i}$ and $b_{2,i}$ are parameters of the second layer for each HSR pixel at fine resolution. The pixel value of LSR LST can be considered as a two-layer linear combination of HSR LSTs [34]. In the practical calculation, Equation (2) can be simplified by merging the two-layer parameters as:

(3) $$LST_{LSR}\left(t\right)=\frac{1}{N}\overset{N}{\underset{i=1}{\sum }}\left(a_{i}LST_{HSR,i}\left(t\right)+b_{i}\right)+\varepsilon \left(t\right)\;$$

Then, pMSRAFM requires at least two pairs of LSR and HSR imageries to solve Equation (3) pixel by pixel within the LSR imagery. That is, the relationship between MODIS and Landsat-8 can be conveniently expressed by Equation (3) in the following matrix form:

(4) $$LST_{LSR}^{MOD}=\frac{1}{n^{2}}\left(\left[\begin{array}{ccc} LST_{1,1}^{Lat} & LST_{1,j}^{Lat} & LST_{1,n}^{Lat}\\ \vdots & \ddots & \vdots \\ LST_{n,1}^{Lat} & LST_{n,j}^{Lat} & LST_{n,n}^{Lat} \end{array}\right]\times \left[\begin{array}{ccc} a_{1,1} & a_{1,j} & a_{1,n}\\ \vdots & \ddots & \vdots \\ a_{n,1} & a_{n,j} & a_{n,n} \end{array}\right]+\left[\begin{array}{ccc} b_{1,1} & b_{1,j} & b_{1,n}\\ \vdots & \ddots & \vdots \\ b_{n,1} & b_{n,j} & b_{n,n} \end{array}\right]\right)+\varepsilon$$

where *n* is the spatial resolution scaling factor between MODIS and Landsat-8, $LST_{i,j}^{Lat}$ is the pixel value at location [i,j] in the Landsat-8 scene, and $LST_{LSR}^{MOD}$ is the MODIS LST pixel value. Equations (3) and (4) show the correspondence between LSTs at different spatial resolutions under ideal conditions. In order to solve Equation (4), the HSR pixels must be classified according to their response characteristics to LST. Furthermore, given the complexity of LST retrieval from Landsat-8 data, we replace the real HSR LST with the physical retrieval algorithm. To keep MODIS and Landsat-8 LST retrieval algorithms consistent, the practical split-window algorithm (SWA) [69,70], as described by Du et al. [65], was selected. The split-window algorithm is well-proven and widely used in LST retrieval from the TIR sensor [71]. Then, the LST value of a Landsat-8 pixel with pixel type *k* can be approximated by SWA as a multivariate quadratic equation:

(5) $$LST_{k}^{SWA}=a_{k,0}+a_{k,1}\left(BT_{i}+BT_{j}\right)+a_{k,2}\left(BT_{i}-BT_{j}\right)+a_{k,3}{\left(BT_{i}-BT_{j}\right)}^{2}+\varepsilon_{SWA}$$

where *a*_*k*,0_, *a*_*k*,1_, *a*_*k*,2_, and *a*_*k*,3_ are equation parameters related to the pixel type *k*. *BT_i_* and *BT_j_* are brightness temperatures converted from the spectral radiance of Landsat-8 band 10 and band 11, respectively. $\varepsilon_{SWA}$ is the adjustment term due to the effects of spatial heterogeneity. For more information about this SWA algorithm, please refer to Du et al. [65]. The LST retrieval algorithm generally relies on ground surface information to characterize the spatial pattern [4]. As the most common environmental factors, NDVI and DEM data can provide complementary information about the spatial variability of LST. They are not only easy to obtain but also directly affect spatial distribution [72]. These two factors are incorporated into the transfer function as covariables to minimize fusion error. Finally, the relationship between MODIS LST and Landsat-8 data can be expressed mathematically by the equation as follows:

(6) $$\begin{array}{ll} LST_{LSR}^{MOD}=\frac{1}{N} & \overset{N}{\underset{i=1}{\sum }}\overset{M}{\underset{k=1}{\sum }}(a_{k,1}\left(BT10_{i}+BT11_{i}\right)+a_{k,2}\left(BT10_{i}-BT11_{i}\right)\\ & +a_{k,3}{\left(BT10_{i}-BT11_{i}\right)}^{2}+a_{k,4}NDVI_{i}+a_{k,5}DEM_{i}+b_{k})+\varepsilon \end{array}$$

where *a*_*k*,1_, *a*_*k*,2_, *a*_*k*,3_, *a*_*k*,4_, *a*_*k*,5_, and *b*_*k*_ are the parameters of the transfer function. *M* is the total number of HSR classifications. The parameters are determined by the least square method. Once the optimal solution is determined, the HSR fused LST for the whole study area can be calculated by the following equation:

(7) $$\begin{array}{ll} LST_{HSR}^{DF}\approx \overset{M}{\underset{k=1}{\sum }} & (a_{k,1}^{\prime }\left(BT10_{k}+BT11_{k}\right)+a_{k,2}^{\prime }\left(BT10_{k}-BT11_{k}\right)\\ & +a_{k,3}^{\prime }{\left(BT10_{k}+BT11_{k}\right)}^{2}+a_{k,4}^{\prime }NDVI+a_{k,5}^{\prime }DEM+b_{k}^{\prime }) \end{array}$$

where $a_{k,1}^{\prime }$, $a_{k,2}^{\prime }$, $a_{k,3}^{\prime }$, $a_{k,4}^{\prime }$, $a_{k,5}^{\prime }$, and $b_{k}^{\prime }$ denote the optimal solution of Equation (6).

#### 2.3.2. Adjustment for HSR Comparison

In order to analyze the relationship between the HSR fused LST and the Landsat-derived LST under the same space-time basis, the value range of the HSR fused LST needs to be adjusted. Since the HSR fused LST aims to preserve the temporal information of MODIS LST and capture the spatial details of the Landsat-8, there is an observational difference between the HSR fused LST and reference LST derived from Landsat. The difference mainly includes two sources: the first is satellite differences in bandwidth, acquisition time, spectral response functions, geolocation errors, and atmospheric correction [73]. The second is the accumulation of simulation errors, which arises from data quality and the rationality of the transfer function. So, it is necessary to adjust the value range of the HSR fused LST for a quantitative analysis of the relationship between the fused LST and Landsat-derived LST. However, it is difficult to accurately estimate the distribution of differences without the corresponding high-resolution reference LST. In this study, we use an indirect method to adjust the discrepancy to fully verify the accuracy of the fusion result. We first calculate the LSR fused LST ($LST_{LSR}^{DF}$) of Landsat and MODIS at low spatial resolution and use it as LSR reference LST. The $LST_{LSR}^{DF}$ is generated based on MODIS emissivity and Landsat-8 brightness temperature according to the single-channel (SC) method [50,74] as follow:

(8) $$LST_{LSR}^{DF}=\frac{BT_{LSR}}{1+\left(\lambda BT_{LSR}/\rho \right)\ln\varepsilon_{LSR}}$$

(9) $$BT_{LSR}=\frac{1}{N}\overset{N}{\underset{i=1}{\sum }}BT_{HSR,i}$$

(10) $$\rho =h\frac{c}{\sigma }\;$$

where $\varepsilon_{LSR}$ is the MODIS Band 31 emissivity. *BT_LSR_* is the average brightness temperature of *N* pixels of Landsat-8 band 10. *λ* is the center wavelength. *σ* is Boltzmann’s constant, 1.38 × 10^−23^ J/K, *h* is Planck’s constant, 6.626 × 10^−34^ J, *c* is the velocity of light. The difference between the HSR fused LST and Landsat-derived LST is estimated by analyzing the relationship between $LST_{LSR}^{DF}$ and MODIS LST $LST_{LSR}^{MODIS}$ as follow:

(11) $$LST_{HSR}^{DF}-LST_{HSR}^{Ref}\propto LST_{LSR}^{MOD}-LST_{LSR}^{DF}\;\;\;$$

**Figure 4:**
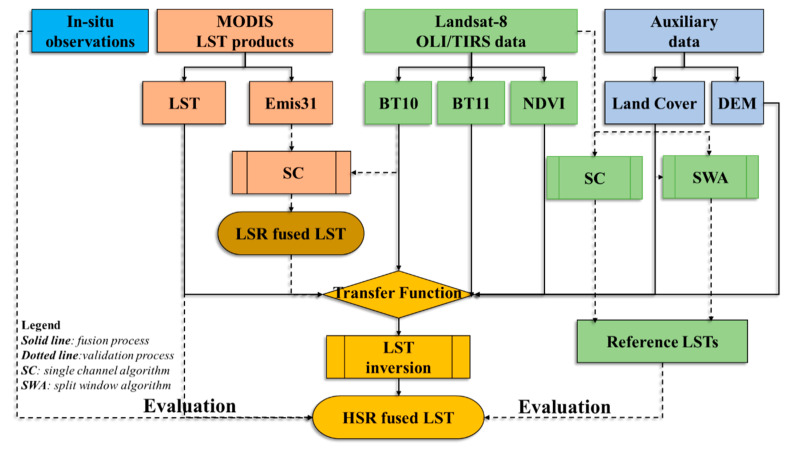
Flowchart of the pixel-based multi-spatial resolution adaptive fusion modeling framework (pMSRAFM) implemented for the LST fusion of Landsat-8 and MODIS data.

To summarize, pMSRAFM is applied to LST fusion based on Landsat-8 and MODIS data. It involves three major steps to solve the transfer function. The first is to calculate brightness temperatures and NDVI from Landsat-8 data. The second step is to extract MODIS LST pixels according to the Landsat-8 scene acquisition date and geolocation. The final step is to determine the parameters of the transfer functions and downscale MODIS LST into Landsat-8 spatial resolution while preserving the original temporal properties. The detailed steps of pMSRAFM implemented for the LST fusion of Landsat-8 and MODIS data are described in Figure 4**.**

#### 2.3.3. Accuracy Assessment

There are many challenges in the evaluation of the satellite-derived LSTs. Compared to the relatively large pixel observed by the satellite, most of the in situ LST observations are measured within a very small area and may introduce significant differences to the satellite measurements [4]. Besides, it is not sufficient to simply compare the fused LST with the ground measurements due to the scarcity and uneven distribution of in situ stations with the lack of observations at satellite overpass time. To fully evaluate the performance of pMSRAFM for generating Landsat-like daily LST, a comprehensive evaluation has been made with three types of reference data. First, $LST_{HSR}^{DF}$ is resampled and compared with MODIS LST product. Second, $LST_{HSR}^{DF}$ is direct pixel-by-pixel comparison with the reference LST derived from Landsat. Finally, the satellite-derived LSTs are compared with the in situ observations. Evaluation metrics included the correlation coefficient (R), *bias*, standard deviation (STD), root mean square error (RMSE), structural similarity index (SSIM) [75], and the index of agreement (d) [76]. *Bias*, *RMSE*, *STD*, and *d* are used to quantitatively validate the difference between the selected LST product and reference LST. *SSIM* is used to describe the closeness of spatial similarity. They are calculated using the following equations:

(12) $$Bias=\frac{1}{S}\overset{S}{\underset{i=1}{\sum }}\left(LST_{i}^{ref}-LST_{i}^{obj}\right)\;\;$$

(13) $$STD=\sqrt{\frac{1}{S-1}\overset{S}{\underset{i=1}{\sum }}\left|LST_{i}^{obj}-LST_{i}^{ref}-\frac{1}{S}\overset{S}{\underset{i=1}{\sum }}\left(LST_{i}^{obj}-LST_{i}^{ref}\right)\right|}\;$$

(14) $$RMSE=\sqrt{\frac{1}{S}\overset{S}{\underset{i=1}{\sum }}{\left(LST_{i}^{obj}-LST_{i}^{ref}\right)}^{2}}\;\;\;\;\;$$

(15) $$d=1-\frac{\Sigma_{i=1}^{S}{\left(LST_{i}^{obj}-LST_{i}^{ref}\right)}^{2}}{\Sigma_{i=1}^{S}{\left(\left|LST_{i}^{obj}-\mu_{ref}\right|+\left|LST_{i}^{ref}-\mu_{ref}\right|\right)}^{2}}$$

where $LST_{i}^{obj}$ is the selected LST to be evaluated, $LST_{i}^{ref}$ is the reference LST. *i* denotes the pixel index. *S* is the number of samples. *µ_ref_* was the mean of *LST^ref^*. The *d* is a standardized measure of the degree of model simulation error and varies between 0 and 1. A value of 1 indicates a perfect match, and 0 indicates no agreement at all [77]. The *SSIM* is a measure of the structural similarity of two images [78,79,80]. It considers similarity degrees of luminance, contrast, and structure of two images [75]. The larger the *SSIM* value, the better, and the maximum is 1. To standardize the LST range, x and y are divided by their maximum value, respectively. Mathematically, the modified *SSIM* is defined as:

(16) $$x=\frac{LST^{obj}}{\max\left(LST^{obj},LST^{ref}\right)},\;y=\frac{LST^{ref}}{\max\left(LST^{obj},LST^{ref}\right)}$$

(17) $$SSIM\left(x,y\right)=\frac{\left(2\mu_{x}\mu_{y}+C_{1}\right)\left(2\sigma_{xy}+C_{2}\right)}{\left(\mu_{x}^{2}+\mu_{y}^{2}+C_{1}\right)\left(\sigma_{x}^{2}+\sigma_{y}^{2}+C_{1}\right)}\;\;\;$$

where *σ* and $\sigma_{xy}$ denote the standard deviation and cross-correlation between *x* and *y*, respectively. *C*_1_ and *C*_2_ are two positive stabilizing constants [80].

## 3. Results

The performance of pMSRAFM was evaluated by comparing the HSR fused LST with MODIS LST products, and the LSTs directly derived from Landsat-8 and in situ observations. The evaluation methods involved visual comparison and statistical indicators. Two pairs of MODIS and Landsat images acquired on 13 September 2015 and 16 July 2017 were used as the primary inputs, named Case 1 and Case 2, respectively. In each case, the MOD11A1 and MOD11_L2 were used to be fused with Landsat-8, respectively. To analyze different types of MODIS data, we conducted a comparative experiment based on MOD11_L2 data, which was divided into two scenarios and labeled “MOD11L2_a” and “MOD11L2_b”. A total of six experiments were conducted to evaluate the performance of pMSRAFM. For visual comparison, all spatial maps were rotated clockwise by 18°. In each case, pMSRAFM shared the same HSR data, but the MODIS data was different. Therefore, we named each experiment with the MODIS LST product name to distinguish each fusion result below.

### 3.1. Fusion Results of Case 1

The LST fusion of MOD11A1 and Landsat-8 was constrained to pixels that: the local time of MOD11A1 pixel observation was limited to 11:42 ~ 11:48 a.m., the zenith angle was within the range from 60° to 63°, and there was no invalid value in the corresponding Landsat pixel block. The final selected pixels accounted for 80.15% of the total number of MODIS pixels. The number of land cover classes was set to 7 according to the FROM-GLC data. The observation time of MOD11L2_a was limited to 10:06~10:12 and the difference with $LST_{LSR}^{DF}$ was less than 0. The final selected pixels accounted for 44.68% of the total. As for MOD11L2_b, the observation time was limited to 11:42~11:48 and the difference with $LST_{LSR}^{DF}$ was greater than 0. The final selected pixels accounted for 36.04% of the total. Because all the fused LSTs shared the same model and auxiliary data, their comparisons with reference LSTs were feasible. The temporal and spatial accuracy of the fusion results in Case 1 were evaluated from the following three aspects:

#### 3.1.1. Comparison with MODIS LST

**Figure 5:**
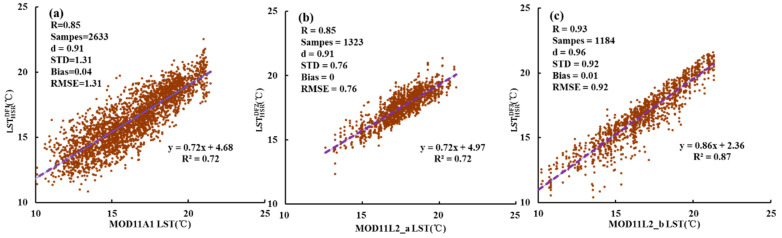
Scatter plots of the HSR fused LST ($LST_{HSR}^{DF}$) against its corresponding MODIS LST product in Case 1 on 13 September 2015. (**a**) MOD11A1 vs $LST_{HSR}^{DF1}$. (**b**) MOD11L2_a vs $LST_{HSR}^{DF2}$. (**c**) MOD11L2_b vs $LST_{HSR}^{DF3}$. The HSR fused LST is resampled to match the spatial resolution of MODIS LST.

A pixel-by-pixel comparison between the HSR fused LSTs and the MODIS LSTs was conducted to quantify the fusion accuracy at the low spatial resolution. To ensure the consistency of spatial resolution, the HSR fused LSTs were aggregated to MODIS-like resolution. Figure 5 illustrates the comparison results between the HSR fused LSTs(y-axis) and their corresponding MODIS LST products (x-axis): Figure 5a $LST_{HSR}^{DF1}$ vs. MOD11A1 LST, Figure 5b $LST_{HSR}^{DF2}$ vs. MOD11L2_a LST, and Figure 5c $LST_{HSR}^{DF3}$ vs. MOD11L2_b LST on 13 September 2015, respectively. The results show an overall good agreement between the HSR fused LSTs and MODIS LSTs with *R^2^* > 0.7 and *d* > 0.9. MOD11A1 has the most samples but the lowest accuracy with *R* = 0.85, *STD* = 1.31, *bias* = 0.04, and *RMSE* =1.31. Figure 5b shows that the difference between the $LST_{HSR}^{DF2}$ and MOD11L2_b is minimal with *RMSE* = 0.76, *bias* = 0, and *d* = 0.91. Most of the evaluation indicators in Figure 5c are the best with *R* = 0.93, *d* = 0.96, and *RMSE* = 0.92, which indicates that the $LST_{HSR}^{DF3}$ retains most of MODIS LST information. MOD11L2 LST has a better precision compared to MOD11A1. Although a few pixels show significant differences, the HSR fused LSTs and MODIS LSTs maintain a high consistency at low spatial resolution.

**Figure 6:**
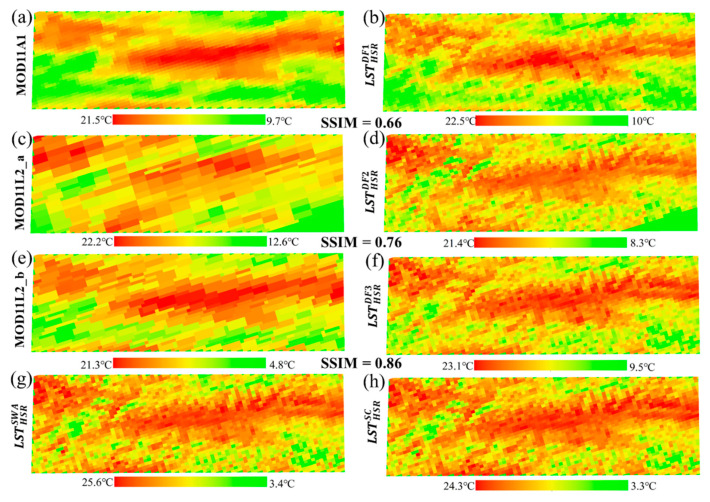
The spatial distributions of the MODIS LSTs (**a**,**c**,**e**), HSR fused LSTs (**b**,**d**,**f**), and reference LSTs (**g**,**h**) of Case 1 on 13 September 2015. The HSR fused LSTs and reference LSTs are resampled to match the spatial resolution of MODIS LST.

The spatial distributions of the HSR fused LSTs and reference LSTs of Case 1 are mapped and shown in Figure 6. Figure 6a,c,e and Figure 6b,d,f are MODIS LSTs and their corresponding HSR fused LSTs, respectively. The SSIM ranges from 0.66 to 0.86, indicating a significant spatial similarity. Figure 6g,h are the resampled reference LSTs. Visually, it can be clearly seen that the spatial pattern of the HSR fused LST is closer to that of reference LST. The HSR LSTs present more delicate spatial details and show a high correlation between them. Figure 5 and Figure 6 indicate that the spatiotemporal information of the MODIS LST is well reflected in the HSR fused LST.

#### 3.1.2. Comparison with Landsat-Derived LST

**Figure 7:**
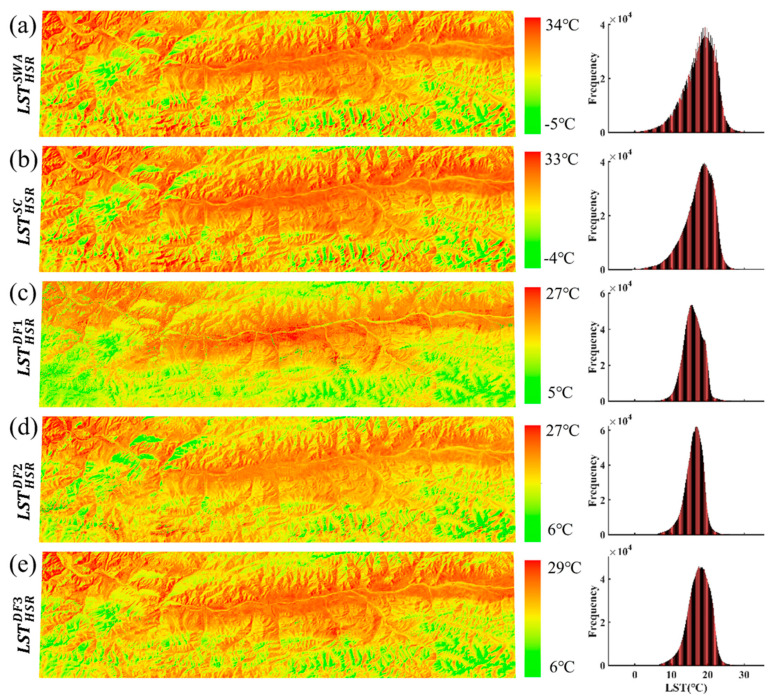
The spatial distribution and frequency histogram of reference LSTs (**a**,**b**) and the fused LSTs (**c**–**e**) in Case 1 on 13 September 2015.

Since the primary goal of pMSRAFM is to improve the spatial resolution of the LSR data meanwhile retaining its temporal information, we pay more attention to the spatial pattern of the HSR fused LSTs at high spatial resolution. The spatial distribution and frequency histogram of the HSR fused LSTs and reference LSTs are shown in Figure 7, and the comparison results are summarized in Table 2. It can be seen from Figure 7 that the LST spatial variations of the five maps are highly similar. Compared with Figure 6, the spatial pattern of the fused LST, especially the fusion result of MOD11L2, is visually improved and more similar to that of the reference LST. The fused LST and reference LST are approximately the same in spatial variation with the terrain. Although the LST ranges (maximum and minimum) are different, the reference LST and fused LST match well in terms of the spatial details. It is observed from the frequency histogram that the reference LST is significantly higher than the fusion LST. Figure 6 and Figure 7 illustrate the fused LST captures the spatial pattern of Landsat LST and retains the original value range of MODIS LST.

As shown in Table 2, *SSIM*, *d*, *bias*, and *R* are improved after fusion, and the best agreement occurs in MOD11L2_b with SSIM ≥ 0.8 and R ≥ 0.9. Figure 7 and Table 2 indicate that the fused LST is substantially the same as the reference LST in the spatial pattern, despite the observational differences. From the perspective of *SSIM* and *d*, the fused LSTs are closer to $LST_{HSR}^{SC}$. The SSIM ranges from 0.77 to 0.88, and the d ranges from 0.79 to 0.94. The $LST_{HSR}^{DF3}$ is the best fused LST in terms of *SSIM* and *d*. It is suggested that pMSRAFM will be practically useful for the LST fusion when the relationship between the paired satellite data can be linearly modeled. Overall, the fusion results of MOD11_L2 are better than that of MOD11A1. Other indicators are also calculated as measures of accuracy. It is found that the *bias* is less than 2 °C, and *RMSE* ranges from 1.7 to 3.5 °C. Further contrastive analysis is discussed in Section 3.3.

#### 3.1.3. Validation with In Situ Observations

**Figure 8:**
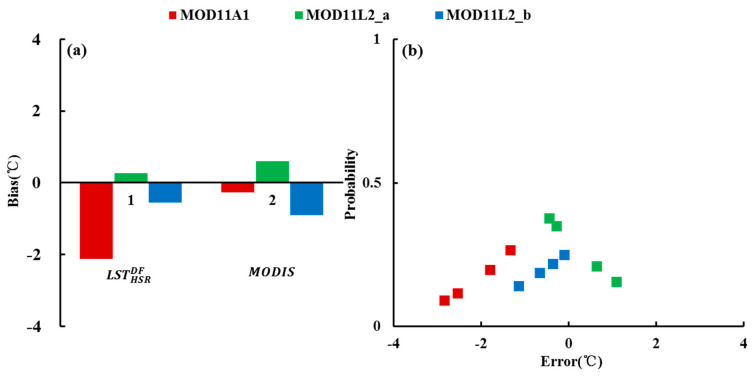
The results of MODIS LST product and its fused LST compared with in situ observations on 13 September 2015. (**a**) bias Histogram and (**b**) fusion error probability distribution.

The HSR fused LST was also compared with in situ observations at the same time. Four stations in the study area were available to evaluate the accuracy of fusion results in Case 1. The error probability was estimated through the Gaussian distribution of the difference between the LSR fused LST and MODIS LST. In addition, the MODIS LSTs and in situ observations were compared to analyze the accuracy under different spatial resolutions. The results are shown in Figure 8. From Figure 8a, the *bias* of the fused LSTs and MODIS LSTs is in the acceptable range (−2.1~0.6 °C). The mean absolute errors between the fused LSTs and in situ observations are 2.1 °C, 0.6 °C, and 0.6 °C, respectively. After fusion, the absolute value of *bias* of MOD11A1 becomes larger, while that of MOD11L2 becomes smaller. Figure 8b shows that the fusion error of MOD11L2 is relatively stable and smaller than that of MOD11A1 LST. The fusion result of MOD11L2_b is the best. By contrast, there is some uncertainty in the fusion result of MOD11A1, which underestimates the real LST by 2 °C. Overall, most of the errors are concentrated in the range −22 °C, so the fusion results are satisfactory. From the probability of error occurrence, the smaller the absolute value of the error, the higher the probability. The maximum probability is 0.38, and the minimum probability is 0.09. The comparisons with in situ observations in Case 1 indicate that the LST generated by pMSRAFM could depict the spatial distribution of the real LST within a reasonable error range.

### 3.2. Fused Results of Case 2

Similar to Case 1, the pixels of each MODIS LST product of Case 2 were also filtered under different constraints. The pixel of MOD11A1 was constrained to that: the local time of MOD11A1 pixel observation was limited to 11:42 ~ 11: 48, the zenith angles were within the range from 60° to 62°, and there was no invalid value in the corresponding Landsat pixel block. The final selected pixels accounted for 85.48% of the total. The number of land cover classes was set to 9 according to the FROM-GLC data. The observation time of MOD11L2_a pixel was limited to 10:06 and the difference with $LST_{LSR}^{DF}$ was less than 0. The final selected pixels accounted for 62.34% of the total. The observation time of MOD11L2_b pixel was limited to 11:42. The final selected pixels accounted for 38.99% of the total. Based on the above dataset, we used pMSRAFM to generate three kinds of daily LST with 30 m resolution to further verify its universality.

#### 3.2.1. Comparison with MODIS LST

**Figure 9:**
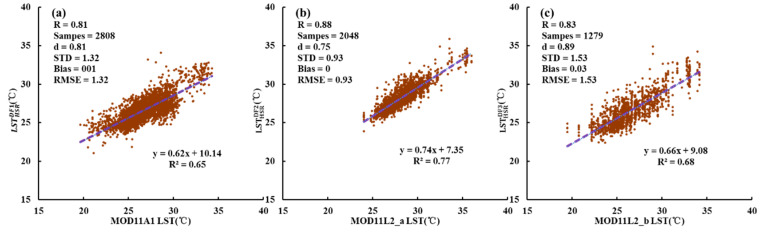
Scatter plots of the HSR fused LST ($LST_{HSR}^{DF}$) against its corresponding MODIS LST product in Case 2 on 16 July 2017. (**a**) MOD11A1 vs $LST_{HSR}^{DF1}$. (**b**) MOD11L2_a vs $LST_{HSR}^{DF2}$. (**c**) MOD11L2_b vs $LST_{HSR}^{DF3}$. The HSR fused LST is resampled to match the spatial resolution of MODIS LST.

**Figure 10:**
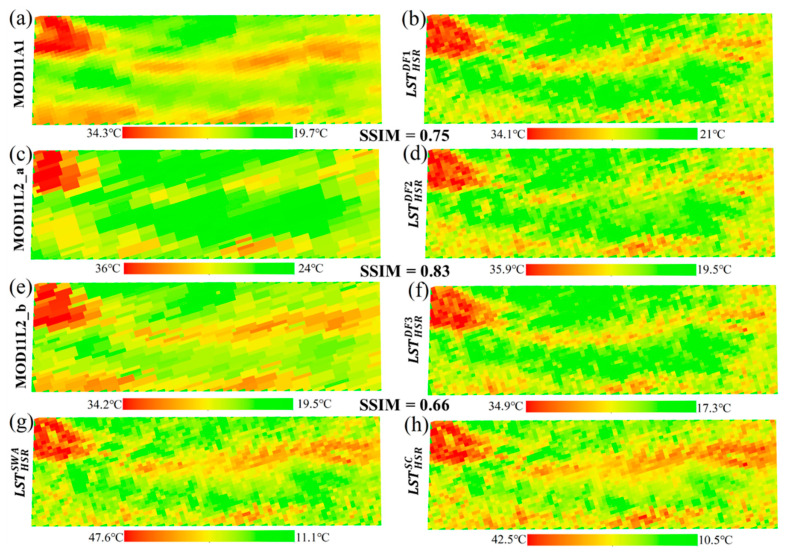
The spatial distributions of the MODIS LSTs (**a**,**c**,**e**), HSR fused LSTs (**b**,**d**,**f**), and reference LSTs (**g**,**h**) of Case 2 on 16 July 2017. The HSR fused LSTs and reference LSTs are resampled to match the spatial resolution of MODIS LST.

Figure 9 shows the pixel-by-pixel comparisons between HSR fused LSTs and MODIS LSTs. As expected, there is a significant correlation between them. The *d* ranges from 0.75 to 0.89, and the range of *R*^2^ is 0.65~0.77. Overall, the *R* is greater than 0.8, *bias* is less than 0.05 °C, and *RMSE* yields a range of 0.93~1.53 °C. The spatial distributions of the fused LSTs and their corresponding MODIS LST products are shown in Figure 10. Figure 10g,h is the resampled reference LSTs. As can be seen from Figure 10, the spatial patterns of the fused LSTs and MODIS LST products are similar, especially in ‘hot’ areas, and the range of *SSIM* is from 0.66 to 0.83. Another proof that the fused LST retains most of the spatial and temporal information of MODIS LST and is closer to the spatial pattern of reference LST at the low spatial resolution.

#### 3.2.2. Comparison with Landsat-Derived LST

**Figure 11:**
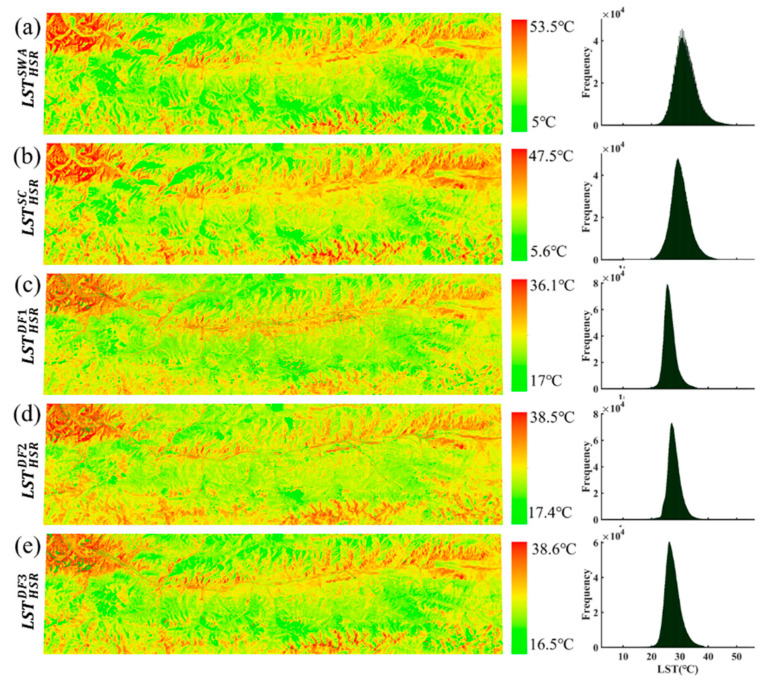
The spatial distribution and frequency histogram of reference LSTs (**a**,**b**) and the fused LSTs (**c**–**e**) in Case 2 on 16 July 2017.

The spatial distributions with frequency histogram of reference LST and the fused LST in Case 2 are shown in Figure 11. As can be seen from the five maps, their spatial patterns are highly coherent and consistent. Intuitively, the spatial pattern of the fused LST is substantially similar to that of the reference LST. Although each LST has a unique value range, most of its data is concentrated in the range of 20 °C ~ 40 °C. Table 3 summarizes the statistical results of the fused LSTs and MODIS LSTs compared with reference LSTs at different spatial resolutions. As expected, pMSRAFM performs well in the spatial pattern simulation of Case 2, in which the *SSIM* ranges from 0.80 to 0.88. Table 3 and Figure 11 show the fused LST tends to share a similar spatial pattern of reference LST derived by the SC algorithm. Again, the fusion result of MOD11L2_b has the best evaluation indicators, and MOD11L2 is better than MOD11A1.

The fused LSTs and MODIS LSTs were compared pixel-by-pixel with the reference LSTs to explore changes in evaluation indicators before and after fusion. As shown in Table 3, MODIS LSTs are also relatively consistent with the reference LSTs generated by the SC algorithm. Indicators for evaluating the spatial pattern are improved by the fusion process. *SSIM*, *d,* and *R* increase by an average of 30%, 19%, and 9%, respectively. *STD*, *bias,* and *RMSE* are partially improved, but not significantly. MODIS LST and fused LST are roughly similar in these indicators. It is found both the average values of *bias* and *RMSE* are above 4 °C at the low spatial resolution before fusion, which indicates Landsat-derived LSTs are 4 °C higher than MODIS LSTs. The average value of *bias* after fusion is 3.7 °C, which is slightly smaller than that of the original MODIS LSTs, suggesting a minor improvement. Furthermore, it indicates that the fused LST not only possess the spatial pattern of Landsat-derived LST but also inherits most of the temporal properties of MODIS LST at the same time. From the above visual comparison and statistical results, pMSRAFM substantially improved the spatial pattern of MODIS LST products with higher accuracy.

#### 3.2.3. Validation with In Situ Observations

**Figure 12:**
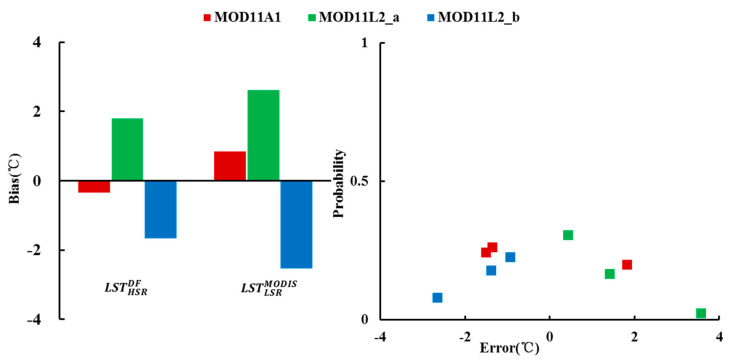
The results of the MODIS LST product and its fused LST compared with in situ observations on 16 July 2017. a: Bias Histogram and, b: fusion error probability distribution.

For Case 2, at the observation time of MODIS LST products, a total of three stations have corresponding observation records that can be used to verify the fusion accuracy. The validation results are shown in Figure 12. Figure 12a shows the *bias* of the HSR fused LSTs and MODIS LSTs against in situ observations. Obviously, the *bias* is distributed between −3 and 3 °C. After fusion, the absolute value of *bias* is reduced by 58%, 31%, and 34%, respectively. The fusion process can reduce *bias* significantly. The relative reduction in *bias* among three experiments indicates that pMSRAFM performs well in the disaggregation of MODIS LSTs. Figure 12b displays the distribution of errors and their corresponding probabilities. It can be seen 78% of fusion errors in Case 2 are within 2 °C, and the mean absolute error is 1.7 °C. The error distribution shows that the larger the absolute value of the error, the lower the probability of occurrence. The results show that compared with the original MODIS LST product, the fused LST can provide more accurate and reliable information.

### 3.3. Comparison with Reference LSTs with Adjustment

The comparison and analysis of fused LST and reference LST derived from Landsat-8 provide a reasonable assessment of the spatial pattern generated by pMSRAFM. Overall, the above comparisons of the two cases show a high degree of consistency. However, uncertainty remains mainly due to differences in LST observations between the two satellites [45]. The orbit parameters of MODIS and Landsat are approximately equal [43], but the data quality, inversion methods, scale effects, and so on will lead to error. Although the spatial similarity can be evaluated by *SSIM* and *R*, the direct comparison pixel values of Landsat-derived LST and fused LST need to be adjusted to reduce the range difference. Theoretically, under clear-sky conditions, the change of LST from 10:00 a.m. to 12:00 a.m. should be approximately a linear upward process. However, these two satellite-derived LSTs did not completely follow this rule in the above two cases (Table 2 and Table 3), in which Landsat-derived LSTs are higher than MODIS LSTs. It is worth mentioning that the *bias* between reference LSTs and fused LSTs is roughly equal to the *bias* between reference LSTs and MODIS LSTs. That means these differences were transferred from LSR LST to HSR LST. It provides an operational way for adjusting the HSR fused LST, making it closer to the HSR reference LST. So, a cross-scale adjustment was performed by using the *bias* of the MODIS LST and $LST_{LSR}^{DF}$ as the observation difference (ΔLST) of reference LST and fused LST. Because ΔLST only changes the range of the HSR fused LST, without affecting the spatial pattern, d, bias, and RMSE were used to re-evaluate the accuracy of fusion results.

The comparisons between reference LSTs and the fused LSTs before and after adjustments are shown in Table 4. It can be found that the reference LSTs are higher than the fused LSTs with the averaged *d* of 0.76, *bias* ranging from −0.03 to 5.42 °C and *RMSE* ranging from 1.69 °C to 6.04 °C. After the adjustment, *d* varies from 0.84 to 0.93, *bias* ranges from −2.0 °C to 2.2 °C, and *RMSE* ranges from 1.6 °C to 3.4 °C. Except for *MOD11L2_b* in Case 1, the differences between reference LSTs and fused LSTs are significantly reduced after the adjustment. In Case 1, the adjustment to MOD11L2_b does not reduce errors as much as that to MOD11A1 and MOD11L2_a, which may be related to the data quality of the LSR pixels involved in the fusion. Even so, its evaluation indicator is slightly better than that of MOD11A1. By contrast, the effect of the adjustment is more pronounced in Case 2, with *d* increased by 15%, *bias* decreased by 84%, and RMSE decreased by 30%. The improvement of these indicators confirms the rationality of cross-scale adjustment and the spatiotemporal reliability of the fused LSTs. It further indicates that pMSRAFM used for LST fusion can achieve similar results as the Landsat-8 LST retrieval algorithm with a relatively high agreement (the average *d* of 0.84).

## 4. Conclusions

This study proposed an operational data fusion framework, named pMSRAFM, for generating the synthetic LST based on the assumption that the correspondence between LSTs at different resolutions or from different sources can be linearly modeled. The synthetic LST was designed to not only preserve the LSR LST temporal information but also has the spatial pattern of HRS LST. The pMSRAFM was implemented and applied to automatically retrieve LST based on Landsat-8 and MODIS data, which presented several improvements over the previous fusion techniques. The most significant improvement is to incorporate an SWA algorithm to establish the linkage between LSR LST and HSR TIR data. Therefore, it only needs a pair of LSR and HSR data, and the HSR data can be reused. This advantage will be significant when pMSRAFM is applied to reconstruct a long-term LST dataset with high spatial resolution. Another merit of pMSRAFM is to address the spatial heterogeneity using the linear spectral mixture analysis. It disaggregates LSR LST by using land cover data as the main distribution factor and BT, NDVI, DEM as covariates. These auxiliary datasets contribute to pMSRAFM obtain more accurate spatial details to deal with surface heterogeneity and reduce fusion errors. Especially the inclusion of HSR BT data is more effective to account for LST variations. Since the fusion processes are executed automatically to fully explore and make the best use of abundant information derived from the existing satellite data, pMSRAFM can be served as an automated data fusion system for regional environmental change research that expects high-quality LST dataset, but lack of ground observations.

In order to evaluate the performance of pMSRAFM, two cases with six experiments were carried out in the Heihe River basin, China. The fusion accuracy was separately analyzed by comparing the fused LSTs with different reference LSTs. Compared with MODS LSTs, the *bias* was approximately equal to 0, and *RMSE* was less than 1.6 °C. When compared with Landsat-derived LSTs, the *SSIM* was greater than 0.8, *d* was higher than 0.74, and *RMSE* was less than 3.4 °C. The errors between the fused LSTs and in situ observations of LST stations within the study area mainly ranged from −2 °C to 2 °C with the average absolute error of 1.3 °C. The comprehensive comparison with MODIS LSTs, Landsat-derived LSTs, and in situ observations proved the rationality and feasibility of pMSRAFM, which could closely capture the spatial pattern of Landsat-derived LST and retain the temporal information of MODIS LST. Besides, the observational differences between these two satellites were fully considered, and the fusion error was estimated by Gaussian distribution. The results revealed that the greater the discrepancy, the lower the probability, and vice versa. Moreover, it also indicated that pMSRAFM could achieve similar results as the Landsat-8 LST retrieval algorithm and the average *d* was 0.84. Hence, the proposed framework provides a practical method for joint retrieval of LST from MODIS and Landsat data, which would be useful for areas lacking in situ observations.

pMSRAFM is not only applicable to the LST fusion of MODIS and Landsat but is also suitable for the data fusion of other LST-related variables to enhance their temporal frequency and spatial resolution. One potential application is to characterize and quantify the spatiotemporal dynamics of regional environment, as well as change detection, target recognition. However, it should be noted that further improvements are needed in three aspects. First, a modular will be introduced to deal with the spatial allocation of fusion error in the follow-up development. Second, subsequent updates will focus more on surface dynamics to improve the reusability of HSR data and predictive ability. Finally, it is essential to incorporate in situ observations into the fusion process for constructing a continuous data series. This study made an initial attempt to develop a multi-source data fusion framework, aiming to generate a more continuous LST series with high spatial resolution. The synthetic products will alleviate the urgent need for timely and accurate monitoring of surface dynamics. In the future, more extensive verification tests will be carried out, with further optimization and improvement of pMSRAFM.

## Figures and Tables

**Table 1:** Geolocation and surface type information of LST stations located in the study area.

Station	Longitude	Latitude	Altitude (m)	Land Cover	Variable/Instrument
A’rou superstation (ARC)	100.46° E	38.05° N	3033	Alpine meadow	Surface temperature/ SI-111(Apogee, USA)
Yakou Station (YKZ)	100.24° E	38.01° N	4148	Alpine meadow	
Jingyangling station (JYL)	101.12° E	37.84° N	3750	Alpine meadow	
E’bao station (EBZ)	100.92° E	37.95° N	3294	Alpine grassland	
A’rou north-facing station (ANF)	100.41° E	37.98° N	3536	Alpine grassland	
A’rou south-facing station (ASF)	100.52° E	38.09° N	3529	Alpine grassland	
Huangzangsi station (HZS)	100.19° E	38.23° N	2612	Farmland	

**Table 2:** The comparison results of the fused LSTs and MODIS LSTs with reference LSTs in Case 1.

LST Product	Reference LST	SSIM	d	STD	Bias	RMSE	R	Sample
**MOD11A1**	$LST_{LSR}^{SWA}$	**0.50**	**0.75**	**2.18**	**1.95**	**2.92**	**0.71**	2633
MOD11A1	$LST_{LSR}^{SC}$	0.51	0.76	2.07	1.68	2.67	0.70	2633
$LST_{HSR}^{DF1}$	$LST_{HSR}^{SWA}$	0.65	0.76	3.00	1.79	3.49	0.73	3,490,550
$LST_{HSR}^{DF1}$	$LST_{HSR}^{SC}$	0.77	0.79	2.66	1.55	3.08	0.76	3,490,550
MOD11L2_a	$LST_{LSR}^{SWA}$	0.69	0.57	1.12	2.71	2.93	0.83	1323
MOD11L2_a	$LST_{LSR}^{SC}$	0.70	0.61	1.01	2.30	2.51	0.82	1323
$LST_{HSR}^{DF2}$	$LST_{HSR}^{SWA}$	0.83	0.84	2.22	1.64	2.76	0.91	3,481,102
$LST_{HSR}^{DF2}$	$LST_{HSR}^{SC}$	0.85	0.87	1.93	1.41	2.39	0.92	3,481,102
MOD11L2_b	$LST_{HSR}^{SWA}$	0.80	0.93	1.48	−0.47	1.55	0.91	1184
MOD11L2_b	$LST_{HSR}^{SC}$	0.81	0.93	1.37	−0.62	1.50	0.90	1184
$LST_{HSR}^{DF3}$	$LST_{HSR}^{SWA}$	0.80	0.92	1.99	−0.20	2.00	0.91	3,495,457
$LST_{HSR}^{DF3}$	$LST_{HSR}^{SC}$	0.88	0.94	1.69	0.03	1.69	0.92	3,495,457

**Table 3:** The comparison results of the fused LSTs and MODIS LSTs with reference LSTs in Case 2.

LST Product	Reference LST	SSIM	d	STD	Bias	RMSE	R	Sample
**MOD11A1**	$LST_{LSR}^{SWA}$	**0.65**	**0.48**	**2.19**	**5.48**	**5.90**	**0.74**	2808
MOD11A1	$LST_{LSR}^{SC}$	0.66	0.59	1.93	3.56	4.06	0.73	2808
$LST_{HSR}^{DF1}$	$LST_{HSR}^{SWA}$	0.80	0.55	2.67	5.42	6.04	0.76	3,473,959
$LST_{HSR}^{DF1}$	$LST_{HSR}^{SC}$	0.84	0.65	2.27	3.58	4.24	0.77	3,473,959
MOD11L2_a	$LST_{LSR}^{SWA}$	0.74	0.49	1.79	4.74	5.07	0.77	2048
MOD11L2_a	$LST_{LSR}^{SC}$	0.77	0.65	1.38	2.75	3.08	0.81	2048
$LST_{HSR}^{DF2}$	$LST_{HSR}^{SWA}$	0.85	0.63	2.54	4.03	4.76	0.78	3,467,619
$LST_{HSR}^{DF2}$	$LST_{HSR}^{SC}$	0.87	0.76	2.07	2.21	3.03	0.81	3,467,619
MOD11L2_b	$LST_{HSR}^{SWA}$	0.58	0.54	2.36	5.39	5.88	0.73	1279
MOD11L2_b	$LST_{HSR}^{SC}$	0.58	0.66	2.13	3.35	3.97	0.72	1279
$LST_{HSR}^{DF3}$	$LST_{HSR}^{SWA}$	0.88	0.67	1.86	4.44	4.82	0.89	3,434,430
$LST_{HSR}^{DF3}$	$LST_{HSR}^{SC}$	0.88	0.79	1.59	2.63	3.08	0.88	3,434,430

**Table 4:** The comparisons between reference LSTs and the fused LSTs before and after adjustments.

No	Fused LST	Reference LST	d	Bias	RMSE	Adjustment			
						ΔLST	*d*	Bias	RMSE
Case1	$LST_{HSR}^{DF1}$	$LST_{HSR}^{SWA}$	0.76	1.79	3.49	−0.89	0.79	0.90	3.13
	$LST_{HSR}^{DF1}$	$LST_{HSR}^{SC}$	0.79	1.55	3.08	−0.89	0.82	0.66	2.74
	$LST_{HSR}^{DF2}$	$LST_{HSR}^{SWA}$	0.84	1.64	2.76	−1.44	0.89	0.20	2.23
	$LST_{HSR}^{DF2}$	$LST_{HSR}^{SC}$	0.87	1.41	2.39	−1.44	0.91	−0.03	1.93
	$LST_{HSR}^{DF3}$	$LST_{HSR}^{SWA}$	0.92	0.20	2.00	−1.37	0.88	1.57	2.54
	$LST_{HSR}^{DF3}$	$LST_{HSR}^{SC}$	0.94	−0.03	1.69	−1.37	0.91	1.33	2.16
Case2	$LST_{HSR}^{DF1}$	$LST_{HSR}^{SWA}$	0.55	5.42	6.04	−5.59	0.80	−0.17	2.68
	$LST_{HSR}^{DF1}$	$LST_{HSR}^{SC}$	0.65	3.58	4.24	−5.59	0.75	−2.01	3.03
	$LST_{HSR}^{DF2}$	$LST_{HSR}^{SWA}$	0.63	4.03	4.76	−1.83	0.74	2.20	3.36
	$LST_{HSR}^{DF2}$	$LST_{HSR}^{SC}$	0.76	2.21	3.03	−1.83	0.86	0.38	2.11
	$LST_{HSR}^{DF3}$	$LST_{HSR}^{SWA}$	0.67	4.44	4.82	−2.54	0.85	1.90	2.66
	$LST_{HSR}^{DF3}$	$LST_{HSR}^{SC}$	0.79	2.63	3.08	−2.54	0.93	0.09	1.59
